# Targeting the orphan nuclear receptor NR2F6 in T cells primes tumors for immune checkpoint therapy

**DOI:** 10.1186/s12964-019-0454-z

**Published:** 2020-01-14

**Authors:** Victoria Klepsch, Maria Pommermayr, Dominik Humer, Natascha Brigo, Natascha Hermann-Kleiter, Gottfried Baier

**Affiliations:** 1 Division of Translational Cell Genetics, Medical University of Innsbruck, Peter Mayr Str. 1a, A-6020 Innsbruck, Austria; 2 Present address: Department of Internal Medicine II, Medical University of Innsbruck, Anichstraße 35, A-6020 Innsbruck, Austria

**Keywords:** Immune checkpoint NR2F6; transcriptional repressor of CD3^+^ effector T cell functions, CRISPR/Cas9 genetically modified cell therapy, Combinatorial treatment regimens

## Abstract

**Background:**

NR2F6 has been proposed as an alternative cancer immune checkpoint in the effector T cell compartment. However, a realistic assessment of the in vivo therapeutic potential of NR2F6 requires acute depletion.

**Methods:**

Employing primary T cells isolated from Cas9-transgenic mice for electroporation of chemically synthesized sgRNA, we established a CRISPR/Cas9-mediated acute knockout protocol of *Nr2f6* in primary mouse T cells.

**Results:**

Analyzing these *Nr2f6*^CRISPR/Cas9 knockout^ T cells, we reproducibly observed a hyper-reactive effector phenotype upon CD3/CD28 stimulation in vitro, highly reminiscent to *Nr2f6*^−/−^ T cells. Importantly, CRISPR/Cas9-mediated *Nr2f6* ablation prior to adoptive cell therapy (ACT) of autologous polyclonal T cells into wild-type tumor-bearing recipient mice in combination with PD-L1 or CTLA-4 tumor immune checkpoint blockade significantly delayed MC38 tumor progression and induced superior survival, thus further validating a T cell-inhibitory function of NR2F6 during tumor progression.

**Conclusions:**

These findings indicate that *Nr2f6*^CRISPR/Cas9 knockout^ T cells are comparable to germline *Nr2f6*^*−/−*^ T cells, a result providing an independent confirmation of the immune checkpoint function of lymphatic NR2F6. Taken together, CRISPR/Cas9-mediated acute *Nr2f6* gene ablation in primary mouse T cells prior to ACT appeared feasible for potentiating established PD-L1 and CTLA-4 blockade therapies, thereby pioneering NR2F6 inhibition as a sensitizing target for augmented tumor regression.

Video abstract.

**Graphical abstract:**

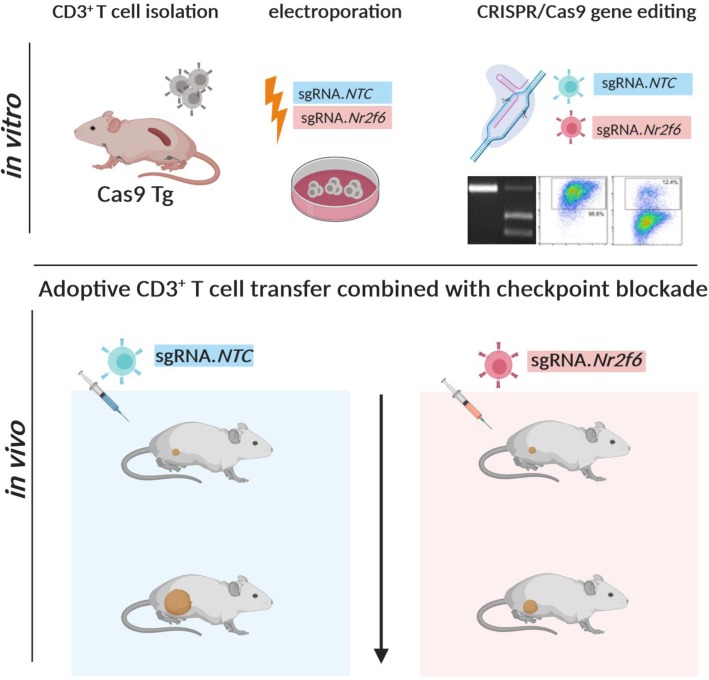

## Background

Solid tumors are infiltrated by effector T cells with the potential to control or reject them; however, the tumor immune microenvironment (TIME) has the ability to restrict the function of these cells at the tumor site and thereby promote tumor growth. An understanding of the interaction between T cells and tumor cells can help unleash the therapeutic anti-tumor activity of effector T cells [1–10]. This concept has led to the successful development of checkpoint blockade immunotherapy targeting either CTLA-4 or PD-1/PD-L1 interaction. These immune checkpoint blockade therapies have proven to be effective in treating several malignancies, including non-small cell lung cancer (NSCLC), renal cell carcinoma (RCC), melanoma, colorectal cancer, head and neck cancer, liver cancer, bladder cancer and Hodgkin’s lymphoma [11–19]. The percentage of responders, however, even though encouraging, is limited, thus highlighting the need for innovative sensitizer approaches mediating improved tumor regression in the clinic.

Particularly, weak tumor-associated inflammatory responses and clinical T-cell dysfunction in inflamed tumors, the latter as a consequence of cancer-mediated immune evasion, remain major hurdles to broader effectiveness of cancer immunotherapy.

Clinical trials suggest that targeting multiple immunosuppressive pathways may better antagonize such tumor immune-therapy resistance and significantly improve patient survival. Therefore, on-going clinical approaches are combining several strategies [20, 21]. Among others, adoptive T cell transfer (ACT) with genetic modifications represents a particularly attractive personalized therapy regimen [22–24], that, in combination with antibody blockade therapy, is likely to achieve more effective remission with long-term tumor control.

Our group identified the orphan nuclear receptor NR2F6 (nuclear receptor subfamily 2, group F, member 6; alias Ear2 and COUP-TFIII) as an intracellular immune checkpoint candidate fine-tuning adaptive immunity [25–30]. *Nr2f6*-deficient mice show an increased tendency for experimentally-induced neuroinflammation [25, 26] as well as improved intratumoral effector T-cell response resulting in strongly decelerated tumor growth in different spontaneous as well as transplantable mouse tumor models [29, 30]. Mechanistically, lymphatic NR2F6 acts as a negative-regulatory signaling intermediate downstream of the antigen receptor and sets the threshold of TCR/CD28 activation-induced effector functions by acting as a transcriptional repressor that directly antagonizes the DNA accessibility of activation-induced NFAT/AP-1 transcription factors at cytokine gene loci such as *Il2* and *Ifng* [29, 30].

Particularly, in light of an advantageous phenotypical effect of a combinatorial PD-L1/NR2F6 inhibition [30], we here explore the concomitant inhibition of these distinct immune checkpoints in the murine MC38 cancer model. In the present work, we have employed ex vivo CRISPR/Cas9-mediated gene ablation of *Nr2f6* prior to therapeutic adoptive transfer, in order to determine whether acute inhibition of NR2F6 gene function indeed enables improved therapeutic anti-cancer activity by the approved PD-L1 or CTLA-4 immune checkpoint therapy in vivo and thus could be a useful dual strategy to elicit meaningful and host-protective tumor immunity.

## Methods

### Mice

*Nr2f6*-deficient mice [29–31] back-crossed eight times on C57BL/6 background were used. The Cas9 transgenic mice were purchased from the Jackson Laboratory, stock no. 028555). Mice were maintained under SPF conditions. All animal experiments were performed in accordance with national and European guidelines and reviewed and authorized by the committee on animal experiments (BMWFW-66.011/0064-WF/V/3b/2016). Investigations were strictly gender-stratified and not blinded. Experimental mice were randomly chosen from litters with a minimal sample size of three.

### Ex vivo T cell analysis

CD3^+^ or CD4^+^ T cells were isolated using either the mouse CD3 or CD4 T Cell Isolation Kit II (Miltenyi Biotec). CD3^+^ or CD4^+^ T cells were activated in complete RPMI medium in the presence of plate-bound mouse 2C11 (αCD3, 5 μg/ml, BioXcell, BE0001–1) and soluble mouse αCD28 (1 μg/ml, BioXcell, BE0015–1). Cells were harvested at the indicated time points.

### T-cell activation and electroporation

CD3^+^ or CD4^+^ T cells from Cas9 transgenic mice were isolated as described above. The procedure of T cell activation, transduction, and analysis was always the same as outlined in Fig. 2a. Isolated T cells were activated with 5 μg/ml 2C11 and 1 μg/ml αCD28 for 2 days. On day 2, cells were electroporated. The optimized electroporation condition for mouse T cells was Amaxa program X01 with the Amaxa™ Mouse T Cell Nucleofector™ kit t from Lonza (VPA-1006). After electroporation, cells were rested overnight in nucleofector media supplemented with 20 ng/ml hIL-2. Next day cells were spread on an anti-CD3 coated 96-well plate with addition of hIL-2.

The CD3 stimulus on day 6 and further cultivated in RPMI solely with hIL-2 until FACS analysis and gDNA isolation was performed.

### sgRNA application

crRNAs for each target gene were purchased from Dharmacon. crRNA target sequences are listed in Table 2. To prepare the crRNAs, they were dissolved in 1x siRNA buffer (100 μM) from Dharmacon and mixed in a 1:1 ratio with tracrRNA to increase stability (crRNA:tracrRNA constructs represent the term sgRNA). The mix was denatured at 95 °C for 5 min and cooled down to anneal at room temperature before stocks were frozen. After annealing, 1 μg of sgRNAs, alone or as a pool of up to five, were electroporated on day 2 after T-cell activation of isolated Cas9 transgenic CD3^+^ or CD4^+^ T cells as described above.

### T7 cleavage assay

Electroporated T cells were harvested at indicated time points, and pellets snap frozen at − 80 °C. gDNA was extracted using the PureLink® Genomic DNA Mini Kit (10053293). The T7 cleavage assay was performed as follows: briefly, targeted regions of CD44 or NR2F6 were PCR-amplified from genomic DNA. The PCR product was denatured and re-annealed in NEBuffer (NEB) using a thermocycler. Hybridized PCR products were digested with T7 Endonuclease I (NEB, M0302S) for 15 min and separated by a 1.5% agarose gel. Primers for PCR are listed in Table 1.

**Table 1:** Sequences of primers for PCR amplification of targeting sites

Primer	Primer Sequences (5′ to 3′)	Amplicon (bp)
CD44.03.F	TGCCTCTGTCCGTCTTCATC	561
CD44.03.R	CCTGTGCACTCTCCATCCTC	
CD44.01.02.F	GAAGTTGCTGGGGTGGGAAT	545
CD44.01.02.R	ACACGGATGGGTTTGCAAAA	
CD44.04.F	GCAGAGTCTGTCTTAGGGTGT	513
CD44.04.R	GCACTGGTTAAAGGAGGGGT	
CD69.01.F	CCCCTCCCAGTGTTTTCTGT	555
CD69.01.R	AGTGCGTTGATGAGGCTTCA	
CD69.02–04.F	TGTTCATGCCCCATCCTGTC	505
CD69.02–04.R	CATACAGTGCTCCCTGCCTC	
Nr2f6.01.05.F	CTCTATCGCCTGGACCTCTG	934
Nr2f6.01.05.F	AGGAAGTTGCCGCAAAACT	580
Nr2f6.01.05.R	GCACGCATGTATCACCAACT	
Nr2f6.02.F	AGGCCAGAGACAGAAGCTGA	764
Nr2f6.02.F	GTGTTTAGGTGGGGCTCTGA	
Nr2f6.03.04.F	TAGCAATGCCCCTCCTATTG	745
Nr2f6.03.04.R	GCCTTTCCAGATACCCATGA	

### Takara kit

The efficiency of sgRNA cleavage was tested on gDNA from wild-type thymocytes with the Guide-it™ sgRNA Screening Kit from Takara (632639). As for the T7 cleavage assay, targeted regions of CD44 or NR2F6 were PCR amplified. The recombinant Cas9 nuclease (500 ng/μl) was added together with sgRNA (50 ng/μl). The cleavage reaction was accomplished following the manufacturer’s instructions and analyzed 1.5% agarose gel.

### Tumor induction

5 × 10^5^ B16-OVA, 5 × 10^5^ MC38 tumor cells (kindly provided by Maximillian Waldner, University of Erlangen, Germany) were injected s.c. into the left flank of 8- to 12-week-old wild-type, or *Nr2f6*^−/−^ mice. The high tumor load was applied to ensure robust tumor growth along with therapy (anti-PD-L1, anti-CTLA-4). Tumor growth was monitored three times a week by measuring tumor length and width. Tumor volume was calculated according to the following equation: ½(length × width^2^). For survival analysis, mice with tumors greater than the length limit of 15 mm were sacrificed and counted as dead. Cell lines were tested negative for mycoplasma (GATC, Konstanz, Germany).

### In vivo antibody blockade

Mice were injected s.c. with 5 × 10^5^ B16-OVA melanoma cells or 5 × 10^5^ MC38 tumor cells and administered either 0.5 mg (B16-OVA) or 0.25 mg (MC38) of anti-mouse PD-L1 (Clone10F.9G2; BE0101), anti-mouse CTLA4 (Clone 9H10, BE0131), corresponding IgG2b (LTF-2; BE0090) or polyclonal Syrian hamster IgG (BE0087) control (all from BioXCell, USA) every 3 days starting from day 3 of tumor challenge according to ref. [30, 32].

### CRISPR/Cas9-mediated *Nr2f6* knockout and adoptive cell transfer

5 × 10^5^ MC38 tumor cells were injected s.c. into C57BL/6 wild-type recipients. Two adoptive cell transfers (ACT) of sgRNA.NTC or sgRNA.Nr2f6.04 electroporated CD3^+^ T cells from Cas9 transgenic mice into wild-type mice were carried out three and 10 days after tumor induction by injecting intra-peritoneally 1 × 10^7^ MACS sorted CD3^+^ T cells (viability > 95%) using the Pan T Cell Isolation Kit II mouse (Miltenyi Biotech 130–095-130). Antibody treatment with 0.25 mg anti-mouse PD-L1 (Clone10F.9G2; BE0101) or anti-mouse CTLA-4 (Clone 9H10, BE0131) with corresponding control antibodies as described above was administered i.p. on day 3, 5, 7, 10, 12 and 14. Tumor growth was subsequently measured as described above.

### Western blotting

Cells were washed and lysed in lysis buffer. Whole-cell extracts were electrophoresed on NuPAGE gels (Invitrogen) and transferred to PVDF membranes. Protein lysates were subjected to immunoblotting with antibodies against αFlag (Sigma, F1804-200UG, 1:1000), and Actin (Santa Cruz Biotechnology Inc., USA: sc-1615, 1:1000).

### Flow Cytometry

Splenocytes or bone marrow cells were depleted of erythrocytes using an erythrocyte lysing buffer and, like lymph node cells or thymocytes, mashed through a 100-μm filter. Splenocytes, thymocytes, lymph node, and bone marrow cells were incubated with FcR Block (BD Biosciences, 553,142) to prevent nonspecific antibody binding before staining with appropriate surface antibodies for 30 min at 4 °C, washed with PBS+ 2% FCS, and used for FACS analysis. For intracellular cytokine staining, cells were stimulated with 50 ng/ml phorbol 12,13-dibutyrate (PDBu, Sigma, P1269), 500 ng ionomycin (Sigma, I0634) and GolgiPlug (BD Biosciences, 555,029) for 4–5 h. After fixation (cytokines: Biolegend fixation buffer (420801), 20 min, 4 °C; transcription factors: eBioscience FoxP3 staining buffer set (Invitrogen, 00–5523-00), > 30 min, 4 °C), cells were permeabilized with the fixation/permeabilization kit (BioLegend, 421,002) for cytokines and the eBioscience Foxp3-staining buffer set (Invitrogen, 00–5523-00) for transcription factors, incubated with FcR Block (BD Biosciences, 553,142) before staining with specific cell surface or intracellular marker antibodies. Data were acquired on a FACSCalibur, or FACS Canto cell analyzer (Becton Dickinson). Data were analyzed using FlowJo software (version 10). The following antibodies were used for flow cytometry: CD4-V500 (BD, 560783), CD4-PE (BD, 553049), CD8a-APC (BD, 553035), CD25-PE (BD, 553866), CD44-PE-Cy7 (Biolegend 103,030), CD62L-APC (BD, 553152), IL-2-APC (BD, 554429), CD8a-PE (eBiosciences, 12–0081-82), IFNγ-PE-Cy7 (eBiosciences, 25–7311-82), CD45-APC (eBiosciences, 17–0451-81), CD3-PE (eBiosciences, 12–0031-83), CD8a-bv421 (BioLegend, 100,738), CD25-bv421 (BioLegend, 102,034), CD69-APC (eBiosciences 17–0691-80), CD11b-PE (eBiosciences, 12–0112-83), Gr1-APC (Biolegend, 108,412), CD19-PE (BD, 557399).

### Statistics

Data were analyzed using Prism 5.03 software (GraphPad Software). Experiments were repeated at least two times with a minimum sample size (*n*) of three. Data are represented as indicated (either the mean ± SEM or ± SD) for all figure panels in which error bars are shown. Overall survival was expressed using the Kaplan−Meier method, and differences between groups were determined using the log-rank test. The *p-*values were assessed using two-tailed unpaired Student’s *t-*test, or two-way ANOVA. A *p* value of < 0.05 was considered statistically significant. *p < 0.05; **p < 0.01; ***p < 0.001.

## Results

### Efficient CRISPR/Cas9-mediated mutagenesis in primary mouse T cells

The CRISPR/Cas9 technology has opened new avenues to physiologically validate alternative and potentially additive and/or synergistic immune checkpoint candidates in preclinical mouse model systems of cancer immunotherapy. In order to avoid the requirement of having to deliver Cas9 to primary mouse T cells, we used the previously established Cas9 transgenic Rosa26-Cas9 mouse line B6(C)-*Gt (ROSA)26Sor*^em1.1(CAG-cas9*,-EGFP)Rsky^/J in which, the Cas9 protein had been linked to eGFP via an internal ribosomal entry site (IRES) under the CAG promoter as described by Chu et al. [33]. Analyzing the Cas9 expression by monitoring bi-cistronic GFP protein levels, recombinant protein expression was confirmed in every hematopoietic subpopulation analyzed, e.g. cells from lymph node (LN), spleen, bone marrow (BM) and thymus (Additional file 1: Figure S1A) as well as the Cas9 protein level on isolated T cells using Western Blot analysis (Additional file 1: Figure S1B). The cellularity of T cells, B cells, and myeloid cells in these secondary immune organs were comparable between wild-type and Cas9 transgenic mice (Additional file 2: Figure S2A-D). Similarly, mice crossed to be homozygous for the Cas9 transgene (double transgenic) displayed no differences in immune cell subset percentages (data not shown) or in the immune cell subset-specific GFP expression (Additional file 1: Figure S1C). In keeping with these observations, the Cas9 transgenic mouse Rosa26-Cas9 on C57/Bl6 background did not show any obvious immune phenotypic abnormalities at least up to the age of 20 weeks tested.

**Fig. 1:**
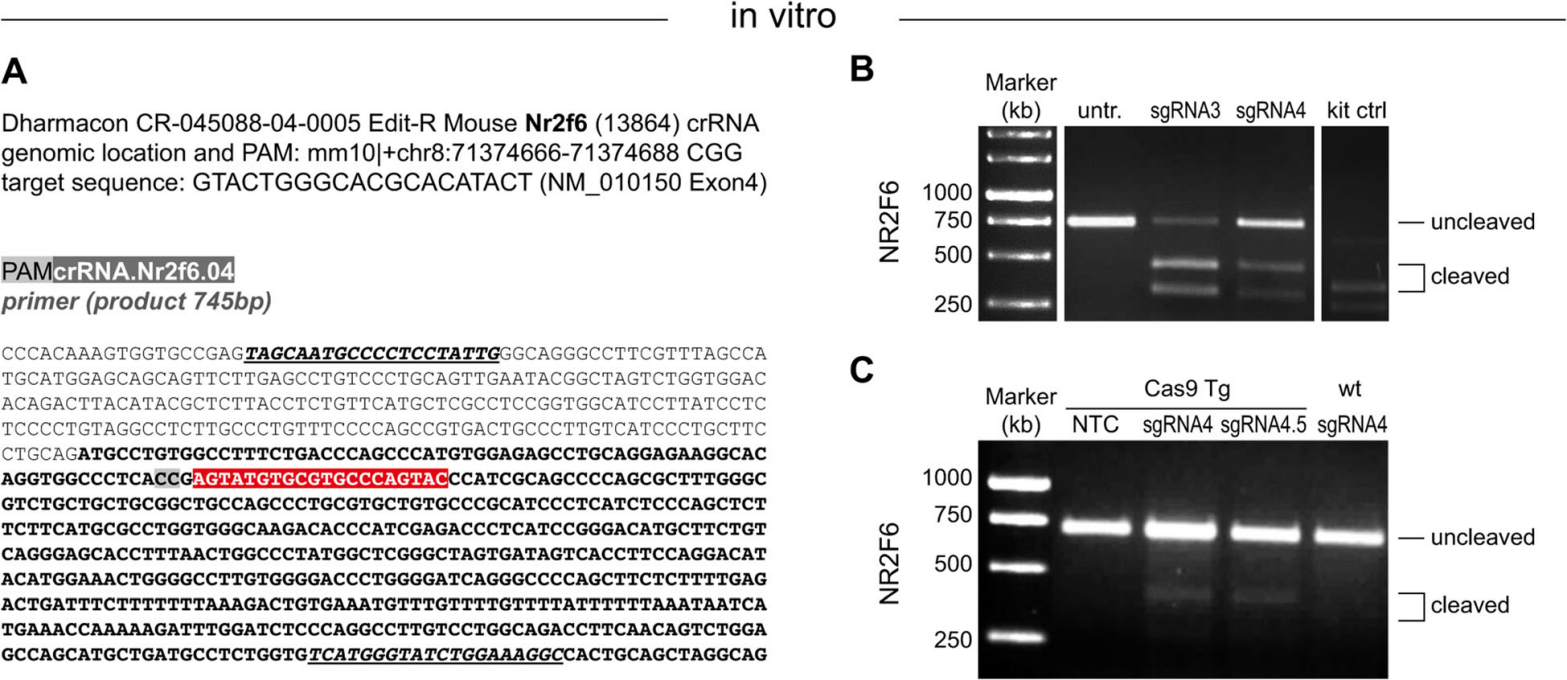
Validation of sgRNAs-mediated knockout targeting NR2F6 in primary lymphocytes. **a** Schematic of the sgRNA-targeting sites within the *Nr2f6* genomic locus. The sgRNA-targeting sequence in red, the protospacer-adjacent motif (PAM) sequence in bold grey and flanking primer pairs (bold, underlined, italics). **b** Detection of sgRNA Cas9-mediated cleavage of *Nr2f6* by PCR with the Takara kit on wildtype thymocytes for sgRNA.Nr2f6.03 and sgRNA.Nr2f6.04 including the kit control and untreated cells without cleavage. **c** T7 cleavage assay on genomic DNA isolated from CD3^+^ Cas9 transgenic T cells using sgRNA.Nr2f6.04 or combining sg. RNA.Nr2f6.04 and sg. RNA.Nr2f6.05 (sgRNA.Nr2f6.4.5) including a non targeting control (NTC) sg. RNA. Results shown are derived from at least two independent experiments. Untr., untreated, NTC, non-targeting control

Whereas primary B cells and dendritic cells have been successfully used for retroviral sgRNA delivery in vitro, high-efficiency transduction needed for primary T cells to directly modify their genomes for functional analysis has not been investigated. Therefore, we established a virus-free delivery protocol of guide RNAs employing electroporation of synthetic sgRNAs into Cas9-expressing T cells. The exon-binding site is exemplarily shown for one sgRNA for NR2F6 (Fig. 1a) with their subsequently designed primer pairs (see Table 1) and sgRNA target sequences (see Table 2). Their cleavage potential was assessed with the Takara kit on isolated genomic DNA sequence amplified by PCR, once the respective sgRNA against NR2F6 and recombinant Cas9 protein was added in vitro (one example is shown in Fig. 1b). A T7 mismatch detection assay has been typically used for the detection and quantification of insertions and deletions (indel) mutations created by the CRISPR/Cas9 system in intact cells [34]. In brief, genomic DNA was isolated from cells 5 days after sgRNA transfection and screened for the presence of site-specific gene modification by PCR amplification of regions surrounding the target sites followed by the T7 cleavage assay. As shown in Fig. 1c, transfection of CD3^+^ T cells from Cas9 transgenic mice with synthetic sgRNA.Nr2f6.04 resulted in detectable indels at the chosen target site as observed by the cleavage bands. This result validated the gene-specific cleavage mediated by CRISPR/Cas9 in primary mouse T cells derived from Cas9 transgenic mice and demonstrated that the selected sgRNAs worked effectively in intact cells.

**Fig. 2:**
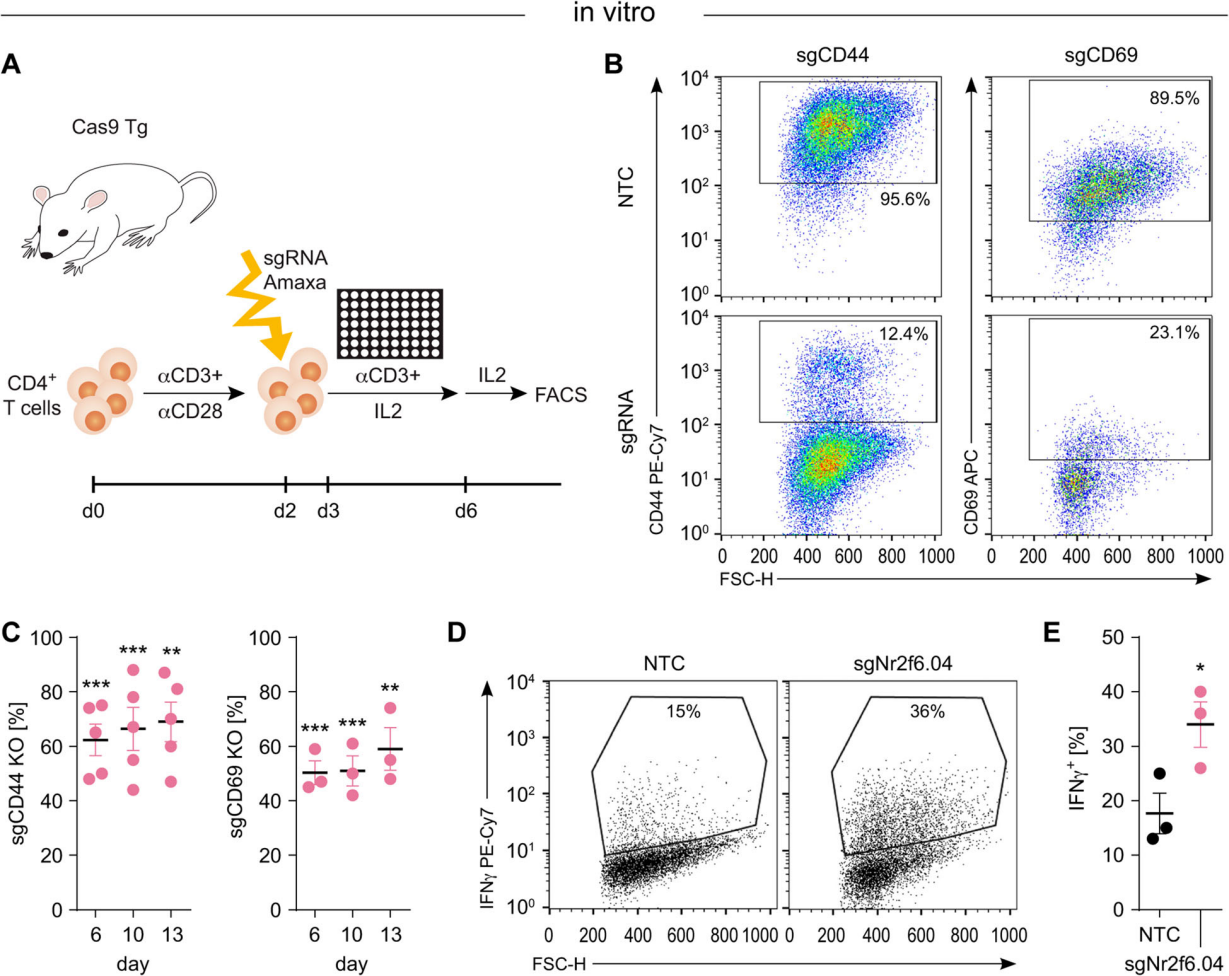
CRISPR/Cas9 mediated gene knockout in primary mouse T cells. **a** Schematic overview of CRISPR/Cas9 mediated gene knockout in isolated CD4^+^ T cells from Cas9 transgenic mice. **b** 10 days post-treatment, flow cytometry assays were performed to measure the loss of CD44 or CD69 in CD4^+^ Cas9 transgenic T cells targeted with sgRNAs against CD44 or CD69. **c** Efficient gene deletion achieved in primary T cells cells following treatment with different sgRNAs. Knockout efficiencies were calculated based on surface marker expression in comparison to NTC treated cells (CD44 KO: d6 *p* = 0.0002, d10 *p* = 0.0009, d13 *p* = 0.0026, CD69 KO: d6 *p* = 0.0003, d10 *p* = 0.0009, d13 *p* = 0.0062). **d** FACS plots and **e** quantification of CD4^+^ T cells with NTC or *Nr2f6* CRISPR/Cas9 mediated knockout on day 10, re-stimulated with PdBU/Ionomycin for 4 h showing enhanced IFNγ cytokine production with *Nr2f6* loss compared to NTC control cells (*p* = 0.0429). NTC, non-targeting control, sgRNA, single guide RNA, Cas9 Tg, Cas9 transgenic. The above experiments are repeated at least two times with similar results. Error bars represent the mean ± SEM

For subsequent experiments, we used this validated sgRNA.Nr2f6.04, which is highly sequence specific within the whole mouse genome as established encoding a perfect match only for the *Nr2f6* gene. To establish the robust efficiency of mouse genome editing using synthetic sgRNA, selected surface marker genes such as CD44 and CD69 have been tested as positive controls in primary mouse CD3^+^ T cells, as they are easily detectable by flow cytometry. Employing this method (Fig. 2a), we reproducibly achieved high efficiencies for bi-allelic gene ablation in primary CD3^+^ T cells derived from the Cas9 transgenic mouse line between 50 and 90% of surface receptors CD44 and CD69 used as convenient positive controls, respectively (sgRNA target sequences in Table 2).

**Table 2:** sgRNA target sequence

Gene	Target Sequence	Order #
Cd44.01	TATGGTAACCGGTCCATCGA	CR-041132-01-0020
Cd44.02	GTCCATCGAAGGAATTGGGT	CR-041132-02-0020
Cd44.03	GTTGGCTGCACAGATAGCGT	CR-041132-03-0020
Cd44.04	TCTGCATCGCGGTCAATAGT	CR-041132-04-0020
Cd44.05	CATGGAATACACCTGCGTAG	CR-041132-05-0020
Cd69.01	ATGTATCCTCGTCATCTGGA	CM-057343-01-0020
Cd69.02	CATTCTTGCAGGTAGCAACA	CM-057343-02-0020
Cd69.03	CATCTTCAGAACAAGAGCGT	CM-057343-03-0020
Cd69.04	GCAATTGTACTTGCCCACTG	CM-057343-04-0020
Cd69.05	CTCCACCACAACCAAGAGTT	CM-057343-05-0020
Nr2f6.01	AATCGTCCTCGGTCGCGCGT	CM-045088-01-0020
Nr2f6.02	GCTGTTCAGCACGGTCGAGT	CM-045088-02-0020
Nr2f6.03	TCCCGGATGAGGGTCTCGAT	CM-045088-03-0020
Nr2f6.04	CTCAAGAAGTGCTTCCGGGT	CM-045088-04-0020
Nr2f6.05	AGGTGTAGCTGAGATTGCGG	CM-045088-05-0020

Transfected T cells were subsequently stimulated, as shown in Fig. 2a and described below: We started with a pool of up to five different crRNAs per gene, these crRNAs were coupled to a tracrRNA to allow guidance and stability and subsequently designated as sgRNAs (crRNA:tracrRNA). We then isolated CD4^+^ T cells from Cas9 transgenic mice and activated them for 2 days with CD3/CD28 crosslinking. On day 2, we performed the electroporation with the sgRNAs, following continued stimulation with αCD3 and IL-2 for three more days. Three days after transfection, we switched to an IL-2 only culture and started FACS analysis of surface marker knockout (Fig. 2b,c) and gDNA extraction for the T7 cleavage assay. For sgRNAs against CD44, a high bi-allelic knockout efficiency of about 70% was reproducibly achieved (Fig. 2b,c). Similar targeting of CD69 resulted in about. 50% of the T cells showing bi-allelic knockout (Fig. 2b,c); however, the viability of CD69^CRISPR/Cas9 knockout^ T cells was reproducibly impaired (data not shown).

This elaborated methodology enabled us to establish acute gene knockouts upon transfection with the given sgRNAs. For the NR2F6 protein, no direct knockout efficiency testing is feasible due to a lack of high-affinity anti-NR2F6 antibodies and low protein expression levels in primary T cells. Instead, we analyzed cytokine production responses from *Nr2f6*^CRISPR/Cas9 knockout^ as an established surrogate marker of NR2F6 function (see [25, 26, 29, 30]) and - reminiscent of germline *Nr2f6*^*−/−*^ T cells - reproducibly observed strongly increased cytokine activation response levels for IL-2 and IFNγ (Fig. 2d, e, and data not shown). This indicates that the CRISPR/Cas9-mediated acute *Nr2f6* gene ablation is effective. Consistent with this observation, acute *Nr2f6* gene editing in mouse T cells reduced the thresholds of antigen receptor signaling and led to hyper-responsiveness of T cells in vitro.

### Germline *Nr2f6* deletion in T cells synergizes with CTLA-4 blockade therapy

**Fig. 3:**
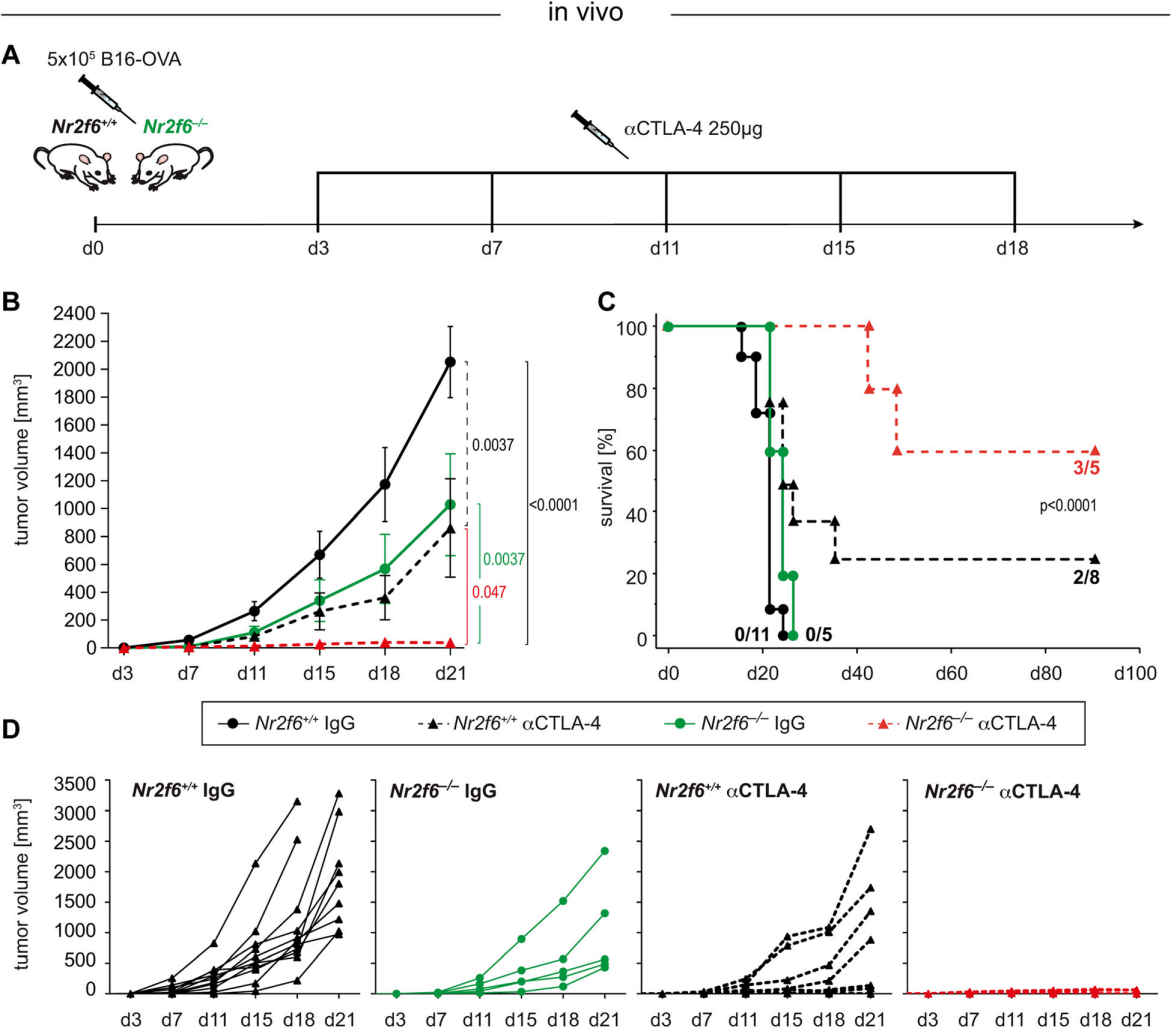
Germline gene ablation of *Nr2f6* in combination with established CTLA-4 immune checkpoint blockade. **a** Schematic overview of tumor injection and immune checkpoint blockade therapy. **b**, **d** Tumor growth curve of wildtype and *Nr26*^*−/−*^ mice that received the high dose of 5 × 10^*5*^ B16-OVA melanoma cells subcutaneously and were treated either with “mono-therapies” of genetic *Nr2f6* deficiency (green, D – second graph, *p* = 0.0037) or CTLA-4 blockade in wildtype mice (dashed black, D – third graph, p = 0.0037) or IgG control antibody (black – wildtype, D – first graph) or with a combination of *Nr2f6* loss and CTLA-4 blockade (dashed red, D – fourth graph, *p* = 0.047). **c** Survival analysis using a Kaplan Meier plot of wildtype and *Nr2f6*^*−/−*^ mice treated either with IgG control or CTLA-4 blocking antibody (*p* < 0.0001) showing 3/5 long-term survivor mice in the combinatorial therapy group of *Nr2f6*^*−/−*^ mice vs. 2/8 survivors in the corresponding control wildtype group. Results shown are derived from at least two independent experiments

In previous studies [28–30] we showed a T cell-intrinsic tumor rejection phenotype by the hyper-reactive effector T cell compartment of whole body *Nr2f6*^*−/−*^ mice using, among others, the transplantable B16-OVA subcutaneous mouse tumor model. Especially in combination with the established immune checkpoint therapy of PD-L1 blockade, we observed a host-protective tumor immunity effect in *Nr2f6-*deficient mice [30]. Additionally, as a therapeutic approach, we previously have demonstrated that adoptively transferred *Nr2f6* siRNA-silenced polyclonal CD3^+^ T cells act as an adjuvant for the established αPD-L1 immune checkpoint in the mouse B16-OVA tumor model [30]. To strengthen our hypothesis of synergy between immune checkpoint therapy and *Nr2f6* inhibition as intracellular immune checkpoint target, we next tested the αCTLA-4 treatment in wild-type and *Nr2f6*^*−/−*^ mice injected with B16-OVA cells showing superior tumor rejection and strongly enhanced survival in the double treatment group (Fig. 3a-d). Of note, 60% long-term survivors were seen in the αCTLA-4 treatment group of *Nr2f6*^*−/−*^ mice (as a combinatorial cancer treatment setting) when compared with 25% in the wild-type monotherapy group (Fig. 3c).

### Acute *Nr2f6* inhibition via CRISPR/Cas9-mediated mutagenesis in T cells is sufficient for priming superior tumor immunity upon CTLA-4 and PD-L1 blockade

**Fig. 4:**
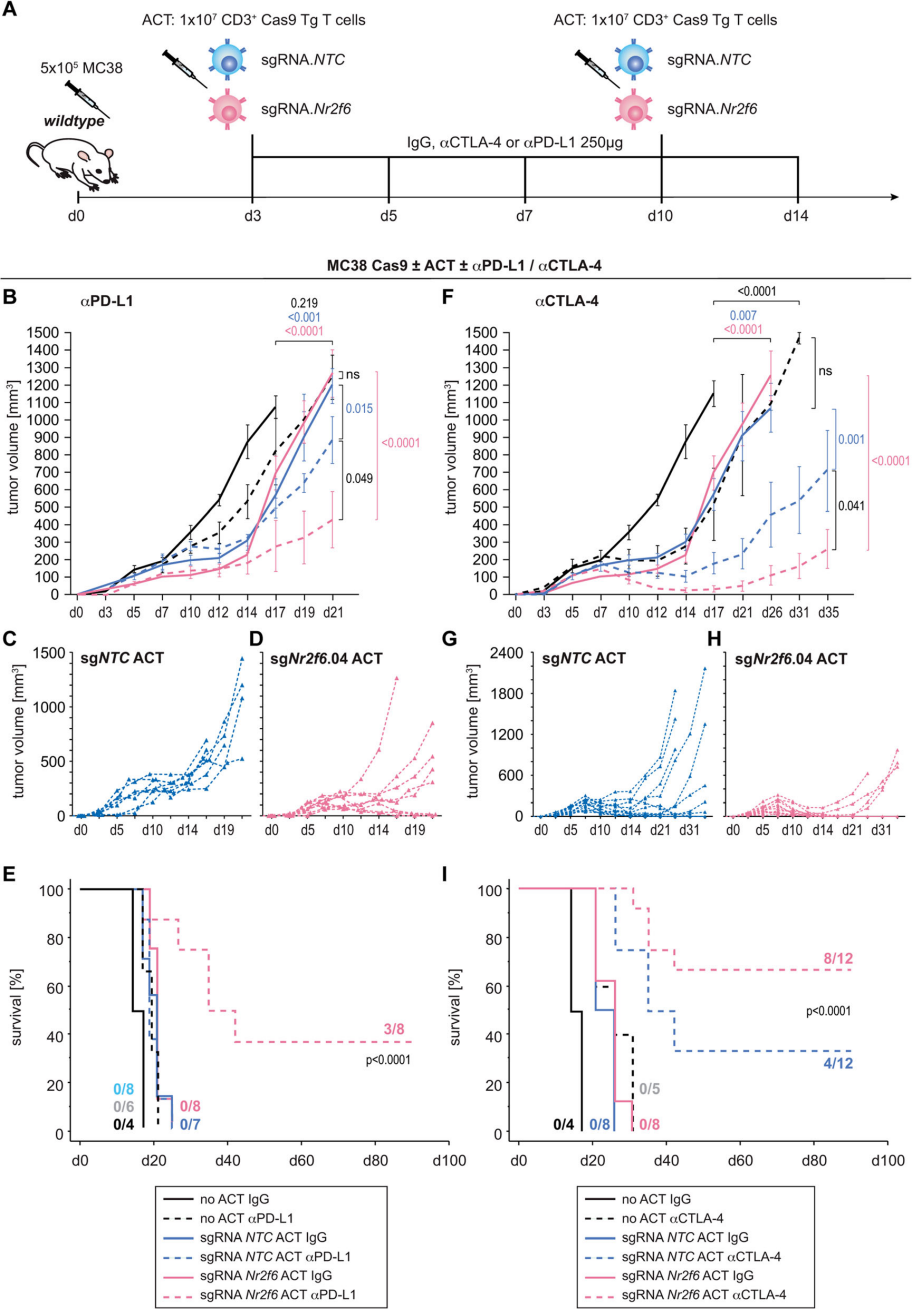
Acute CRISPR/Cas9 mediated gene ablation of *Nr2f6* prior therapeutic adoptive transfer in combination with established CTLA-4 and PD-L1 immune checkpoint blockade. **a** Experimental scheme of tumor injection (d0), adoptive cell transfer therapy of CRISPR/Cas9 mediated *Nr2f6* gene knockout CD3^+^ T cells (d3 and d10) and immune checkpoint blockade therapy (d3, d5, d7, d10, d14). **b** Tumor growth curve of wildtype mice injected with 5 × 10^5^ MC38 tumor cells, treated with αPD-L1 (dashed lines) or IgG2b control antibody (continuous lines) in combination with no ACT (black), ACT with CD3^*CRISPR.NTC*^ (blue, **c**) or ACT with CD3^*CRISPR.Nr2f6*^. (pink, **d**) CD3^+^T cells. **f** Tumor growth curve of wildtype mice injected with 5 × 10^5^ MC38 tumor cells, treated with αCTLA-4 (dashed lines) or IgG control antibody (continuous lines) in combination with no ACT (black), ACT with CD3^*CRISPR.NTC*^ (blue, **g**) or ACT with CD3^*CRISPR.Nr2f6*^. (pink, **h**) CD3^+^ T cells. **e** Survival analysis using a Kaplan Meier plot of wildtype mice treated with αPD-L1 resulting in 3/8 long-term survivor mice in the combinatorial therapy group with an ACT of CD3^*CRISPR.Nr2f6*^ T cells (*p* < 0.0001). **i** Kaplan Meier analysis of wildtype mice treated with αCTLA-4, resulting in 8/12 long-term survivor mice in the combinatorial therapy group with an ACT of CD3^*Crispr.Nr2f6*^ T cells vs. 4/12 survivors in the corresponding control CD3^*Crispr.NTC*^ ACT group. Results shown are derived from at least two independent experiments

With the ultimate goal of developing an innovative immunotherapy-based combinatorial approach, we aimed to confirm our hypothesis that NR2F6 inhibition could vastly enhance T cell effector responses specific for tumor antigens as well as confer protection from the immunosuppressive TIME in a relevant murine tumor model system. Particularly because there is a significant correlation of lymphatic PD-1 or CTLA-4 with lymphatic NR2F6 expression in human NSCLC patients [30], we wanted to determine whether acute *Nr2f6* inactivation enables anti-cancer activity in vivo. Pursuing this concept, and mirroring in principle a pharmacological treatment, therapeutic adoptive transfer (ACT) of acute *Nr2f6* gene-edited autologous CD3^+^ T cells into tumor-bearing mice in a combinatorial CTLA-4 or PD-L1 blockade setting was carried out (Fig. 4a). Fully immunocompetent wild-type mice received a high dose of MC38 tumor cells and were treated with therapeutic ACT twice on days 3 and 10 using CD3^+^ T cells from Cas9 transgenic mice transfected with control sgRNA (sg. RNA.NTC) or sgRNA.Nr2f6.04, in combination with either αPD-L1 (Fig. 4b-e) or αCTLA-4 (Fig. 4f-i) checkpoint blockade, respectively. Therapeutic adoptive transfer of *Nr2f6*^*CRISPR/Cas9 knockout*^ polyclonal CD3^+^ T cells was sufficient for causing a significant delay in tumor growth in this dual treatment setting when compared to mice receiving CD3^*CRISPR.NTC*^ control cells (Fig. 4b-f). As a remarkable result, 66.67% or 8 out of 12 mice receiving CD3^*CRISPR.Nr2f6*^ with αCTLA-4 (Fig. 4h, i) and 37.5% (3/8) with αPD-L1 (Fig. 4d, e) therapy survived the tumor burden. With αPD-L1 treatment alone, none of the control mice survived (Fig. 4c, e), whereas one-third or 4 out of 12 of mice receiving control ACTs treated with αCTLA-4 (Fig. 4g, i) survived the tumor challenge. Thus, these data provide an independent confirmation of the critical NR2F6 function in T cell-mediated cancer immunity, strongly suggesting that in combination with either of the approved PD-L1 and CTLA-4-targeted immune checkpoint therapies, T cell-based ACT therapies have increased efficacy from modulation of the NR2F6 inhibitory signaling pathway.

Taken together, adoptively transferred *Nr2f6*^CRISPR/Cas9 knockout^ CD3^+^ T cells act as a robust “sensitizer” for both the established αPD-L1 and αCTLA-4 immune checkpoint blockade in the mouse MC38 tumor model, significantly improving immune-activating cancer therapy outcomes.

## Discussion

The physiological relevance of NR2F6 function in clinically relevant cancer models as well as in T cell biology has been firmly established [29, 30, 36–38]. Employing CRISPR/Cas9-mediated mutagenesis technology in primary T cells, we here provide strong pre-clinical evidence that acute manipulation of lymphatic NR2F6 similarly elicits superior anti-cancer immune responses in combination with established checkpoint blockade. Employing a robust sgRNA transfection-based knockout system into primary mouse T cells from Cas9 transgenic mice, efficient CRISPR-mediated *Nr2f6* gene editing for cancer immunotherapeutic purpose has been established. It has previously been shown that *Nr2f6*^*−/−*^ T cells are hyper-reactive in regard of cytokine production (IL-2, IFNγ, TNFα) as those cytokines are direct target genes of NR2F6-dependent transcriptional repression [25, 26, 28–30] leading to an improved anti-tumor immune contexture at the tumor site [29, 30]. Consistent with this working hypothesis, the phenotype of *Nr2f6*^CRISPR/Cas9 knockout^ T cells - as an acute genetic loss-of-function approach employed in this study – similarly induced hyper-responsiveness, thereby resembling the germline *Nr2f6* knockout immune phenotype in the effector T cell compartment. Of note, siRNA-mediated *NR2F6* silencing in human T cells similarly reduced the thresholds of antigen receptor signaling and induced hyper-responsiveness in polyclonal T cells ([30] and Fig. 2d, e).

Tumor cells upregulate the expression of PD-L1, which indicates the induction of adaptive resistance. Our previous data even indicated that PD-1 is strongly upregulated in *Nr2f6*-deficient T cells and that a combinatorial blockade of both the PD-L1/PD-1 and the NR2F6 pathways is effective in delaying tumor growth and improving long-term survival with complete tumor regression [30]. Next, in order to validate our working hypothesis that NR2F6 inhibition could vastly enhance T-cell effector responses specific for tumor antigens in vivo as well as confer protection from the immunosuppressive TIME in a relevant preclinical murine tumor model system, acute *Nr2f6* gene editing in combination with the established immune checkpoint blockade was performed. In the present study, we investigated whether blockade of PD-L1 together with acute CRISPR/Cas9-mediated *Nr2f6* deletion can cause rejection of tumors that otherwise do not respond to anti-PD-L1 monotherapy. This was also investigated with the combination of CTLA-4 blockade/acute CRISPR/Cas9-mediated *Nr2f6* deletion. Remarkably, we found that blockade of either PD-L1 or CTLA-4 upon ACT of *Nr2f6*^CRISPR/Cas9 knockout^ CD3^+^ T cells decelerated tumor growth. Strikingly, we discovered that complete regression of established tumors could be achieved in 37.5% of mice using combined αPD-L1 therapy and in 66.67% of mice with combined αCTLA-4 therapy (Fig. 4e, i). Thus, combining adoptive transfer of *Nr2f6* CRISPR/Cas9 genetically modified T cells showed synergistic effects with both the established PD-L1 and the CTLA-4 checkpoint blockade to promote tumor regression and increase survival in a subcutaneous tumor mouse model.

Taken together, this data suggest that disruption of lymphatic Nr2f6 converts tumor-infiltrating T cells into IFNγ- and IL-2-hypersecreting effector cells, apparently sufficient to prime the TIME for immune checkpoint therapy to more effectively control tumor growth. NR2F6 has been defined as a negative master switch of both central nervous system inflammation [25–27], on the one hand, and anti-tumour responses, on the other [29, 30]. Remarkably and despite the improved clinical outcome in whole body *Nr2f6-/-* tumour-bearing mice subjected to PD-L1 blocking (combinatorial NR2F6/PD-L1 inhibition group) when directly compared to wild-type mice under mono-therapy, no exacerbated signs of systemic immune-related adverse effects (irAE) such as tissue immune cell infiltrates, colon length or weight change after anti-PD-L1 treatment in *Nr2f6*-deficient mice were observed during a follow-up period of 3 months ([30] and data not shown). This suggests that side effects of NR2F6 inhibition might not hamper the potential of a therapeutic approach targeting lmyphatic NR2F6. Of further note, nuclear receptors have a long history of successful drug discovery [28, 36, 39]. Since NR2F6 is an orphan nuclear receptor with no valid information about endogenous ligands; however, new therapeutic avenues pharmacologically targeting NR2F6 will only be successful once a small molecule ligand has been identified. Along this line of argumentation, genetic deletion of both or even just one allele of the *Nr2f6* gene [30] initiates tumor control. This haploinsufficiency of the *Nr2f6* gene function observed in heterozygous *Nr2f6+/-* mice further highlights the suitability of pharmacologically targeting NR2F6 in clinical treatment regimens in the future.

At a time when monoclonal antibodies targeting the PD-1/PD-L1 or CTLA-4 pathways are dominating the immunotherapy field, and despite some challenges that remain, optimism is currently high that the use of gene-editing technology is breaking new ground. Notably, our preclinical proof of concept study on the acute and CRISPR/Cas9-mediated *Nr2f6* gene depletion acts as a robust “sensitizer” for established immune checkpoint blockade in the mouse MC38 tumor model. The envisioned process to maximize the efficacy of gene-modified human T cell-based ACT will involve drawing autologous T cells from the patient’s blood through apheresis, electroporating them with pre-assembled sgRNA-Cas9 ribonucleoproteins (sgRNA-Cas9 RNPs) ex vivo to simultaneously disrupt chosen target genes, e.g. *NR2F6* and potentially other immune-regulatory genes, prior to re-infusion. Such immune augmentation or sensitizing is envisioned as a way forward to extend the benefits of clinical immune-oncology therapies to a larger number of cancer patients. In terms of individualized adoptive therapy of NR2F6 gene-modified human T cells, the unique feature of lymphatic NR2F6 as an alternative intracellular immune checkpoint may influence combinatorial cancer therapies in the future. 

## Conclusion

In summary, these findings are in line with our previous data from germ-line knockout studies and indicate that the *Nr2f6*^CRISPR/Cas9 knockout^ T cells are comparable to germline *Nr2f6*-deficient T cells, a result providing an independent confirmation of the cancer immune checkpoint function of lymphatic NR2F6. As a pre-clinical proof of concept, this establishes NR2F6 as a promising cancer therapeutic candidate target and NR2F6 inhibition as a sensitizing concept for next-generation immune-oncology regimens. From a clinical perspective, if valid, such combinatorial immunotherapy regimens including *NR2F6* gene-edited ACTs are likely to strengthen the portfolio of precision medicine applications for the successful development of personalized cancer immune therapy for improving patient survival.

## Supplementary information

**Additional file 1:** **Figure S1** Characterization of Cas9 transgenic mice. (A) FACS analysis of GFP expression in CD4^+^ T cells, CD19^+^ B cells, Gr1^+^ granulocytes, and CD11b^+^ monocytes derived from the Cas9 transgenic mouse on a B6 background [33]. Cells were isolated from LN (first column), spleen (middle) or thymus/BM (third column). Cells from wildtype C57Bl/6 mice (grey) and Cas9 transgenic mice (pink) are shown in the same FACS plots. (B) Western blot analysis of lysates prepared from isolated CD3^+^ T cells of wildtype or Cas9 transgenic mice using a Flag antibody. (C) Representative histogram of GFP expression in wildtype, single transgenic Cas9, or double transgenic Cas9 mice.

**Additional file 2:** **Figure S2** Comparison of Cas9 and wildtype mice in regard of immune cell subsets. Percentages of the indicated immune cell populations within all cells in the lymph node (A), the bone marrow (B), the spleen (C), and the thymus (D) of wildtype (black) or Cas9 transgenic mice (pink). Each mouse is represented by one dot. Results shown are derived from two independent experiments. (A-D) Results reach no statistical significance.

## Data Availability

The datasets used and/or analysed during the current study are available from the corresponding author on reasonable request.

## Abbreviations

ACT: Adoptive cell transfer
BM: Bone marrow
bp: base pairs
Cas9: CRISPR associated protein 9
CD: Cluster of differentiation
CRISPR: Clustered regularly interspaced, short palindromic repeats
crRNA: CRISPR RNA
CTLA-4: Cytotoxic T lymphocyte associated antigen 4
d: day
FACS: Fluorescence-activated cell sorting
gDNA: genomic DNA
GFP: Green fluorescent protein
IFNγ: Interferon gamma
IL: Interleukin
KO: Knock-out
LN: Lymph node
NR2F6: Nuclear receptor subfamily 2 group F member 6
NRs: Nuclear receptors
NTC: Non-targeting control
PAM: Protospacer adjacent motif
PCR: Polymerase chain reaction
PD-1: Programmed cell death 1
PD-L1: Programmed cell death-ligand 1
sgRNA: single - guide RNA
TCR: T cell receptor
TIME: Tumor immune microenvironment
tracrRNA: trans-activating RNA
WT: Wild-type
