# Supplementary figures and images for: Targeting the orphan nuclear receptor NR2F6 in T cells primes tumors for immune checkpoint therapy
Source: Cell Commun Signal. 2020 Jan 14;18:8. doi: 10.1186/s12964-019-0454-z (PMC6961368; doi:10.1186/s12964-019-0454-z)

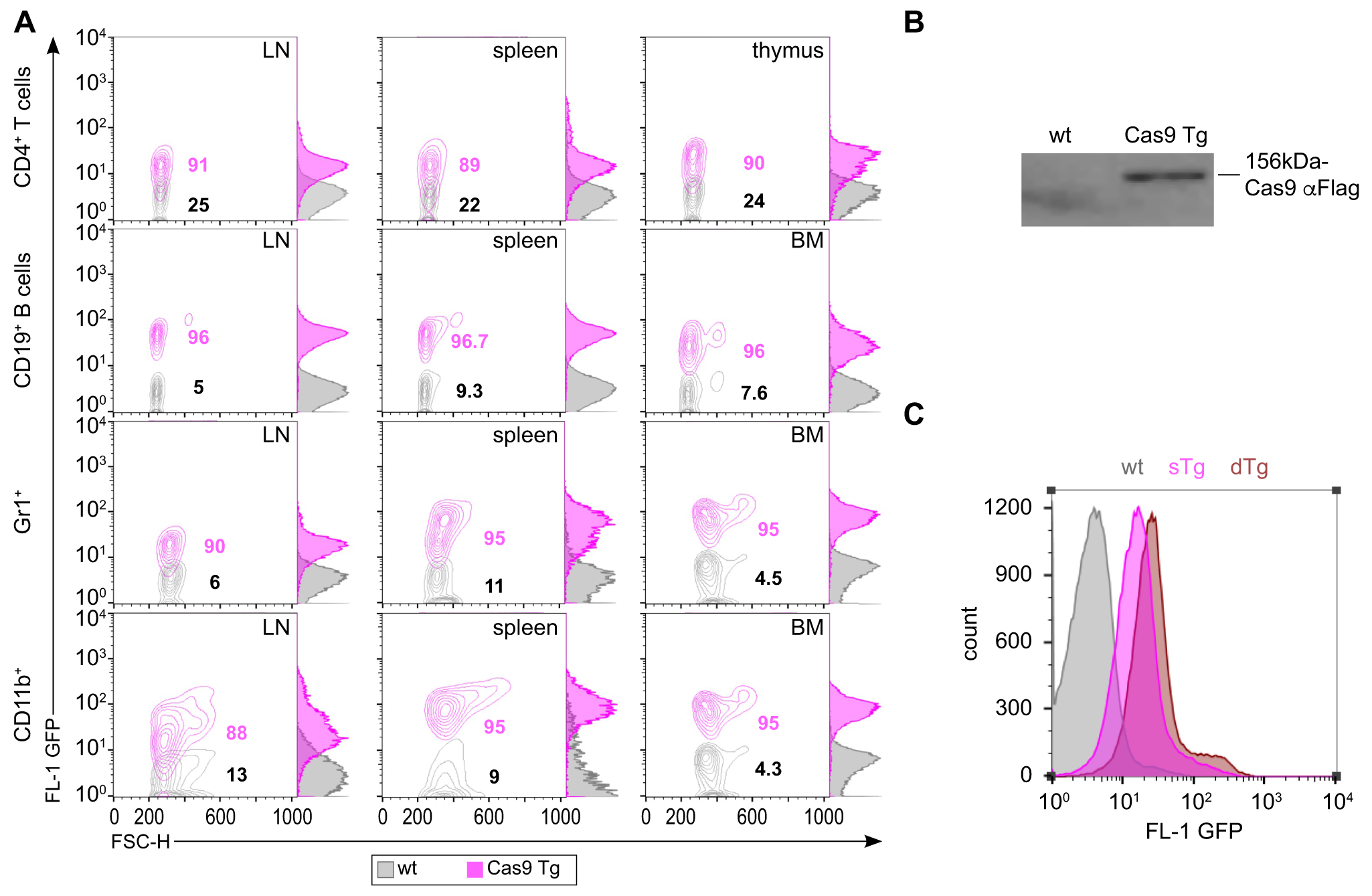

Supplement: Supplementary file 2 — Additional file 1: Figure S1 Characterization of Cas9 transgenic mice. (A) FACS analysis of GFP expression in CD4+ T cells, CD19+ B cells, Gr1+ granulocytes, and CD11b+ monocytes derived from the Cas9 transgenic mouse on a B6 background [33]. Cells were isolated from LN (first column), spleen (middle) or thymus/BM (third column). Cells from wildtype C57Bl/6 mice (grey) and Cas9 transgenic mice (pink) are shown in the same FACS plots. (B) Western blot analysis of lysates prepared from isolated CD3+ T cells of wildtype or Cas9 transgenic mice using a Flag antibody. (C) Representative histogram of GFP expression in wildtype, single transgenic Cas9, or double transgenic Cas9 mice. [file 12964_2019_454_MOESM2_ESM.tif]

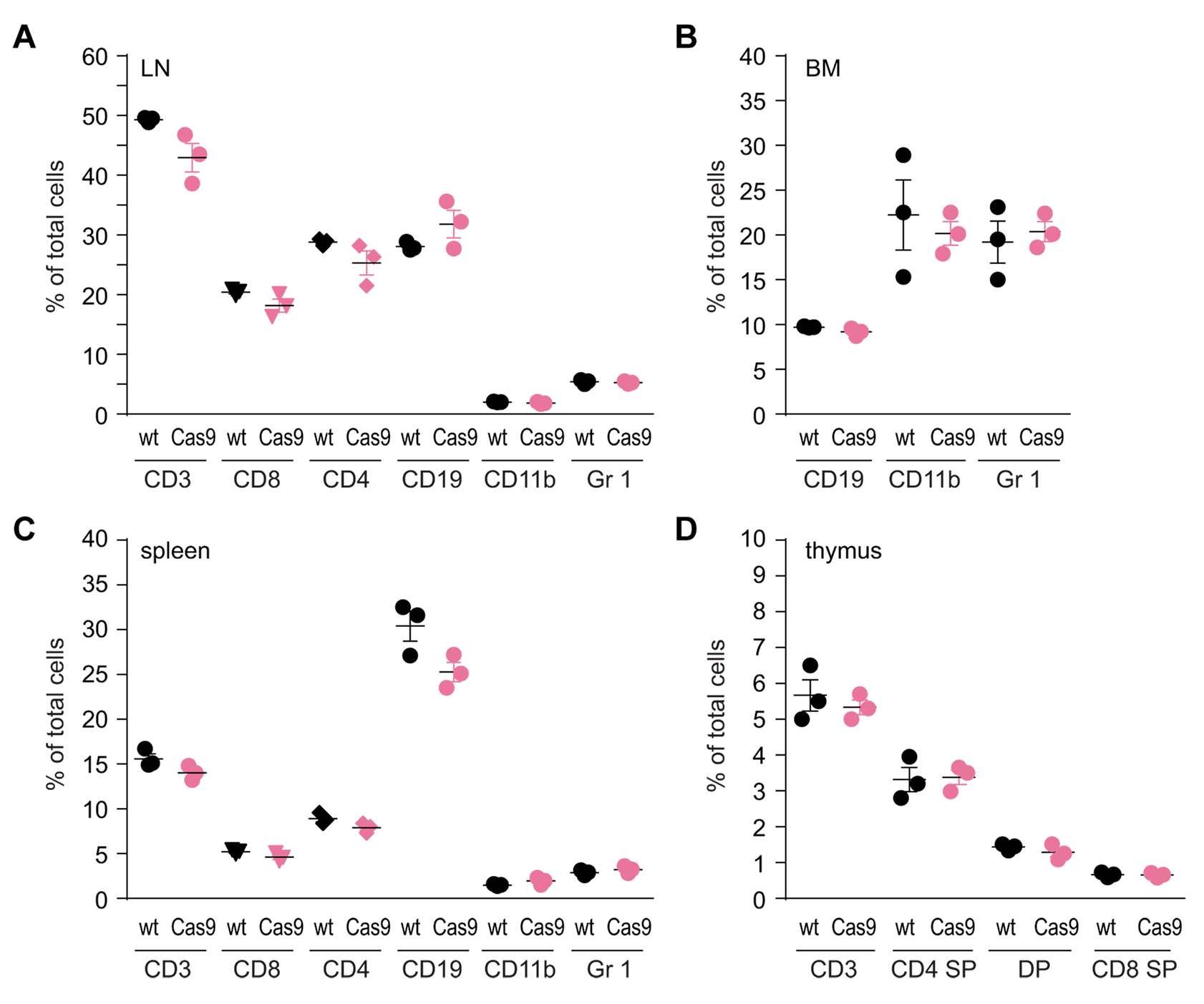

Supplement: Supplementary file 3 — Additional file 2: Figure S2 Comparison of Cas9 and wildtype mice in regard of immune cell subsets. Percentages of the indicated immune cell populations within all cells in the lymph node (A), the bone marrow (B), the spleen (C), and the thymus (D) of wildtype (black) or Cas9 transgenic mice (pink). Each mouse is represented by one dot. Results shown are derived from two independent experiments. (A-D) Results reach no statistical significance. [file 12964_2019_454_MOESM3_ESM.tif]
